# Inhibition of O-GlcNAcylation Reduces Cell Viability and Autophagy and Increases Sensitivity to Chemotherapeutic Temozolomide in Glioblastoma

**DOI:** 10.3390/cancers15194740

**Published:** 2023-09-27

**Authors:** Amanda V. Leonel, Frederico Alisson-Silva, Ronan C. M. Santos, Rodrigo P. Silva-Aguiar, Julia C. Gomes, Gabriel M. C. Longo, Bruna M. Faria, Mariana S. Siqueira, Miria G. Pereira, Andreia Vasconcelos-dos-Santos, Luciana B. Chiarini, Chad Slawson, Celso Caruso-Neves, Luciana Romão, Leonardo H. Travassos, Katia Carneiro, Adriane R. Todeschini, Wagner B. Dias

**Affiliations:** 1 Instituto de Biofísica Carlos Chagas Filho, Universidade Federal do Rio de Janeiro, Rio de Janeiro 21941-902, RJ, Brazil; amandavl@biof.ufrj.br (A.V.L.); caruso@biof.ufrj.br (C.C.-N.); leo.travassos@biof.ufrj.br (L.H.T.); adrianet@biof.ufrj.br (A.R.T.); 2 Instituto de Microbiologia Paulo de Góes, Universidade Federal do Rio de Janeiro, Rio de Janeiro 21941-902, RJ, Brazil; 3 Departamento de Genética, Instituto de Biologia, Universidade Federal do Rio de Janeiro, Rio de Janeiro 21941-590, RJ, Brazil; 4 Instituto de Ciências Biomédicas (ICB), Universidade Federal do Rio de Janeiro, Rio de Janeiro 21941-902, RJ, Brazilluromao@gmail.com (L.R.); kcarneiro@histo.ufrj.br (K.C.); 5 Department of Biochemistry and Molecular Biology, University of Kansas Medical Center, Kansas City, KS 66103, USA

**Keywords:** O-GlcNAc, Glioblastoma (GB), cancer, O-GlcNAcylation, HBP, autophagy

## Abstract

**Simple Summary:**

Glioblastoma (GB) is the most common and aggressive type of malignant brain tumor; however, despite advances in treatment modalities, it remains largely incurable. Current therapeutic protocols are ineffective, and temozolomide (TMZ), the main chemotherapy used in GB treatment, has a high resistance rate. Aberrant O-GlcNAcylation is related to the tumorigenesis of several tumor types, and targeting O-GlcNAc transferase (OGT) is a possible therapeutic target for some tumor types. Here, we investigated the effect of OGT inhibition on cellular proliferation, cell death, and autophagy, as well as whether it could improve the effect of TMZ on cell viability. Our findings indicated that targeting OGT shows promising potential as a therapeutic strategy for treating GB.

**Abstract:**

Glioblastoma (GB) is the most aggressive primary malignant brain tumor and is associated with short survival. O-GlcNAcylation is an intracellular glycosylation that regulates protein function, enzymatic activity, protein stability, and subcellular localization. Aberrant O-GlcNAcylation is related to the tumorigenesis of different tumors, and mounting evidence supports O-GlcNAc transferase (OGT) as a potential therapeutic target. Here, we used two human GB cell lines alongside primary human astrocytes as a non-tumoral control to investigate the role of O-GlcNAcylation in cell proliferation, cell cycle, autophagy, and cell death. We observed that hyper O-GlcNAcylation promoted increased cellular proliferation, independent of alterations in the cell cycle, through the activation of autophagy. On the other hand, hypo O-GlcNAcylation inhibited autophagy, promoted cell death by apoptosis, and reduced cell proliferation. In addition, the decrease in O-GlcNAcylation sensitized GB cells to the chemotherapeutic temozolomide (TMZ) without affecting human astrocytes. Combined, these results indicated a role for O-GlcNAcylation in governing cell proliferation, autophagy, cell death, and TMZ response, thereby indicating possible therapeutic implications for treating GB. These findings pave the way for further research and the development of novel treatment approaches which may contribute to improved outcomes and increased survival rates for patients facing this challenging disease.

## 1. Introduction

Glioblastoma (GB), a grade IV astrocytoma, is considered the most common malignant primary brain tumor, accounting for approximately 15% of all primary neoplasms of the brain and central nervous system [1,2,3]. Sadly, patients affected by this pathology have an average life expectancy of just 15 months from diagnosis of the disease to death [3]. The standard therapy is based on maximum resection surgery followed by radiotherapy and chemotherapy using the alkylating agent temozolomide (TMZ) with adjuvant treatment [4,5,6]. Despite these efforts, GB remains highly resistant to treatment, often leading to tumor recurrence [7]. Therefore, there is still an urgent and pressing need to develop new therapeutic strategies [8,9,10,11]. Recent studies have highlighted the potential of targeting enzymes involved in cell metabolism as a promising approach in the pursuit of effective therapies against GB. Such a targeted approach offers hope for developing treatments that could overcome the current challenges in managing this aggressive brain tumor [11,12]. Cancer cells, including those in glioblastomas (GB), elevate their intake of glucose and glutamine, which are fundamental for the synthesis of UDP-GlcNAc. The first step of this pathway is catalyzed by the enzyme glutamine fructose-6-phosphate amidotransferase (GFAT) [12]. UDP-GlcNAc serves as a pivotal substrate that is crucial for protein O-GlcNAcylation [13]. O-GlcNAcylation occurs on the serine and threonine residues of nuclear, cytoplasmic, and mitochondrial proteins [13]. The enzyme O-GlcNAc transferase (OGT) catalyzes the addition of GlcNAc to proteins, whereas the enzyme O-GlcNAcase (OGA) removes the sugar. O-GlcNAcylation changes can modulate protein function, such as enzyme activity, subcellular localization, stability, or transcription [14]. Aberrant O-GlcNAcylation and the altered protein expression of OGT and OGA are associated with poor prognoses and tumor grades in some cancer types [15,16]. 

O-GlcNAcylation can regulate a wide range of cellular functions that are important for tumorigenesis including cellular proliferation, cell cycle, cell death, autophagy, and chemotherapy resistance [17,18]. The pharmacological inhibitor of OGT (Osmi-1) synergistically increases the apoptosis induced by the chemotherapy drug Doxorubicin in HepG2 cells [19]. Necrosis and autophagy are also affected by O-GlcNAcylation [20,21,22]. Furthermore, studies evaluating the impact of O-GlcNAcylation on autophagy and its development on tumorigenesis or tumor progression have already been carried out on bladder cancer [23,24]. Autophagy acts as one of the mechanisms of therapeutic resistance and is correlated with tumor proliferation and survival [25,26]. Glioblastoma stem cells present autophagy as a mechanism of chemoresistance and radioresistance that can cause tumor regrowth, making the current standard treatment protocols mostly unsuccessful [27,28]. In a past study, a decrease in O-GlcNAcylation activated autophagic degradation in healthy astrocytes, but the positive modulation of O-GlcNAc did not promote the alteration of autophagic flow [29]. 

Here, we showed increases in O-GlcNAcylation, OGT, and GFAT levels in GB cells when compared with non-tumoral astrocytes. The pharmacological inhibition of OGA induced hyper O-GlcNAcylation, activating autophagy and promoting cell survival. On the other hand, the inhibition of OGT decreased O-GlcNAcylation levels and inhibited autophagy, activating cell death by apoptosis. In addition, OGT inhibition increased the sensitivity of cells to the chemotherapeutic drug TMZ without reducing the viability of human astrocytes. Combined, our data suggested that OGT is a promising target for GB therapy.

## 2. Materials and Methods

### 2.1. GB Cell Lines and Human Astrocytes

We used the commercial U87MG cell line and GBM11, a more malignant cell line isolated from a tumor recurrence [30], as well as untransformed human astrocytes cells as controls. The human astrocyte cells were obtained through temporal lobe biopsies on epileptic patients, as described in [31]. The use of human astrocyte cells was performed with patient consent, and the procedures were reviewed and approved by the Ethics Committee of the Ministry of Health of Brazil.

The cell lines were sustained using Dulbecco’s Modified Eagle Medium (DMEM) (DMEM Powder, pyruvate 12800017) (Gibco, Darmstadt, Germany) enriched with 10% heat-inactivated Fetal Bovine Serum (FBS) (Sigma-Aldrich, St Louis, MO, USA), along with 100 units of penicillin and 100 mg/mL streptomycin (Sigma-Aldrich, St Louis, MO, USA). These cultures were maintained under a humidified environment containing 5% CO_2_ at 37 °C. In this study, human astrocytes up to the third passage were employed.

### 2.2. Cell Line Observation

The U87MG and GBM11 cells were observed using phase-contrast microscopy for the evaluation of cell density and morphology. The treatment conditions were as follows: the vehicle control (dimethyl sulfoxide (DMSO)), 1 μM Thiamet G (TMG), 200 μM TMZ, and 25 μM Osmi-1 (all from Sigma-Aldrich, St Louis, MO, USA), with or without TMZ, for 24 h. The cells were photographed with an inverted microscope (Axiovert 135) (Zeiss, Jena, Germany) using the 10× objective.

### 2.3. Obtaining the U87MG GFP-LC3+ and Immunofluorescence Microscopy

To generate the U87MG GFP-LC3+ cells, we used 2–3 mL of the supernatant of the HEK cells containing the retrovirus to transform the U87MG cells, as described in [32]. The effectiveness of the transformation was observed under a fluorescence microscope, and the cells were expanded in 2 flasks of 75 cm^2^ for the later selection of the GFP+ cells through sorting. For the autophagy assay, 1 × 105 cells/mL of the U87MG GFP-LC3+ cells were plated in glass coverslips inside a 24-well plate. On the following day, the glioblastoma cells were treated in triplicate with or without 1 μM TMG or 25 μM Osmi-1 alone or in combination with 20 μM chloroquine (CQ), an autophagy inhibitor that was added for the last 4 h of the 24 h period of incubation. The cells were fixed with 4% paraformaldehyde for 15 min at room temperature and washed twice in 1x PBS. Then, the cells were permeabilized in 0.1% Triton X-100 for 5 min, washed twice with PBS, and incubated with DAPI (4,6-diamidino2-phenylindole; Sigma-Aldrich, St Louis, MO, USA) 1:1000 to mark the nuclei for 10 min. Finally, the cells were washed twice with PBS and subsequently mounted on glass slides. The images were acquired using a Zeiss Cell Observer Yokogawa microscope-spinning disk using the 1000× objective.

### 2.4. Transmission Electron Microscopy of the Ultrathin Sections

Cells of the U87MG cell line with or without the treatments of 1 μM TMG or 25 μM Osmi-1 for 24 h were fixed in 2.5% glutaraldehyde in 0.1 M of sodium phosphate buffer (pH of 7.2) for 1 h at room temperature, washed 3 times in 0.1 M of sodium cacodylate buffer (pH of 7.2), and post-fixed in the same buffer plus 1% osmium tetroxide + 0.8% potassium ferrocyanide + 5 mM calcium chloride for 45 min in the dark. The samples were washed 3 more times in 0.1 M sodium cacodylate buffer (pH of 7.2), dehydrated in an increasing series of acetone (30, 50, 70, 90, and 100%) for 10 min each, and infiltrated in Epon resin: acetone (1: 1) for 16 h. Then, the samples were added to pure resin for 6–9 h at room temperature and stored to polymerize for 48 h at 60 °C. Ultrathin sections of the material (60–70 nm) were obtained using a Leica EM UC7 ultramicrotome and contrasted with 5% uranyl acetate in water for 45 min, followed by lead citrate for 5 min [33], and examined under a JEOL 1200 electron microscope operating at 80 kV.

### 2.5. Immunoblotting 

For the Western blotting, as described in [34], the cells were washed twice with PBS and then subjected to 150 µL RIPA lysis buffer (50 mM Tris-HCL (pH of 7.4), 150 mM NaCl, 1% Triton X-100, 100 mM EDTA, 0.25% sodium deoxycholate, 0.1% SDS, 1 mM PMSF, and 1:100 Halt protease inhibitor cocktail (Pierce; Perbio, Cramlington, UK). The protein concentrations of the samples were measured using a BCA protein assay kit (Pierce™, Thermo Scientific). We used 25 μg of the whole-cell protein extracts to be separated by SDS-PAGE gels. The following antibodies were used: OGT, OGA, GFAT, O-GlcNAc (CTD110.6), and β-actin (Cell Signaling Technology, Danvers, MA, USA and O-GlcNAcylation antibody (RL2), obtained from Sigma (St. Louis, MO, USA). Chemiluminescent detection was performed using an ECL kit (GE Healthcare, Pittsburgh, PA, USA), and densitometry was performed using ImageJ 1.5.3.

### 2.6. Cell Cycle and Annexin V Assay

For the cell cycle analysis, the cells were trypsinized, washed with PBS (pH of 7.4), centrifuged at 1500 rpm for 3 min, and resuspended in 100 µL of FACS Buffer (PBS 1x (pH of 7.4) + 5% SFB + 0.1% NaN3) containing 100 μg/mL Ribonuclease A (RNAse) + 0.2% Saponin. The cells were maintained in this buffer for 20 min at room temperature and then incubated with 50 µg/mL propidium iodide (PI) for DNA staining. For DNA content evaluation, 10,000 events were acquired and analyzed on an FACSCalibur flow cytometer (BD Bioscience) for cell distribution at different stages of the cell cycle according to the DNA contents (G1, S, and G2/M). FlowJo v10 was used to analyze the distribution of phases G0/G1, S, and G2/M. The experiments were completed in triplicate.

The apoptotic and necrotic cells were measured using Annexin V conjugated with allophicocyanin (APC Annexin V BioLegend, Amsterdam, Netherlands) and PI staining (to detect the necrosis cells). The cells were resuspended in 100 µL of FACS buffer containing 5 µL of Annexin V (1:20) + 50 µg/mL PI iodide and incubated for 15 min before analyzing the samples by flow cytometry. In the assay, 10,000 events were acquired per condition, and the readings were carried out on channels FL-4 for Annexin V and FL-2 for PI. FlowJo v10 software was used for the data analysis. The experiments were completed in triplicate.

### 2.7. Ki-67 Flow Cytometry Staining Protocol

The GBM11 cells treated with 25 μM Osmi-1 or DMSO for 24 h, and then they were dissociated with trypsin, washed with PBS containing 2% FBS, centrifuged at 1500 RPM for 5 min at 4 °C, and resuspended in 1 mL of FACS buffer (PBS + FBS). In a 96-well plate, 5 × 105 cells were fixed and permeabilized with 100 μL of BD Cytofix/Cytoperm™ solution (BD cat. 554714) for 20 min at 4 °C, and then they were washed 2 times with BD Perm/Wash™ buffer (BD cat. 554723). Next, we proceeded with the incubation with the Ki-67 primary antibody (BD cat. 550609) diluted in BD Perm/Wash™ buffer (1:200) for 20 min at 4 °C in the dark, followed by 2 rounds of washing as described above. Then, the cells were incubated with the FITC anti-IgG mouse secondary antibody diluted in BD Perm/Wash™ buffer (1:200) for 20 min at 4 °C in the dark, and again, they were washed as described above. Finally, the cells were resuspended in 300 μL of FACS buffer and analyzed using a BD LSRFortessa™ X-20 cell analyzer.

### 2.8. Trypan Blue Assay and MTT Assay

The human astrocyte cells were incubated with a vehicle solution (DMSO) or Osmi-1 in different concentrations (10 μM, 25 μM, 35 μM, and 50 μM) for further analysis of the cell viability and proliferation by 3-(4,5-dimethylthiazol-2-yl)-2,5-diphenyltetrazoliumm (MTT) and trypan blue exclusion assays. To investigate the effects of TMG and Osmi-1 on cell proliferation, the U87MG and GBM11 cells were treated with or without 1 μM TMG or 25 μM Osmi-1 for 24 h, and after the incubation interval of each experiment, the cells were trypsinized and 10 μL of trypan blue (Sigma-Aldrich) was added, after which we counted them with a Neubauer camera. To investigate the influence of Osmi-1 with TMZ, we treated the U87MG cells with a concentration from 100–800 μM of TMZ in the presence or absence of 25 μM of Osmi-1 for 24 h and then performed an MTT assay. Each experimental condition was performed in triplicate, and the experiments were repeated at least three times. 

### 2.9. Growth Kinetics of 3D Cellular Spheroids 

We generated the 3D spheroid growth assays as described in [35], and flat-bottom 96-well plates were coated with 2% agarose diluted in water and left at 4 °C for 15 min for agarose solidification. The U87MG cells were plated at 3000 cells per well in DMEM medium containing 10% FBS. The 96-well plates were subjected to 400× *g* centrifugation for 5 min, and the plates were then incubated in a humidified incubator at 37 °C and 5% CO_2_ for 72 h. The subsequent experiments were initiated with spheroid diameters of between 300 and 400 µm. 

We analyzed the growth of the spheroids by quantifying the volumes over 7 days from day 0, when the 3D cultures were incubated with the drugs. The fully formed three-dimensional structures (day 0) derived from the U87MG cells were treated with 1 µM TMG, 25 µM Osmi-1, and 200 µM TMZ or with 25µM Osmi-1 and 200 µM TMZ. As a control, 0.2% DMSO was used. The properly formed spheres were photographed in an inverted microscope (Axiovert 135) (Zeiss, Jena, Germany) using the 4× objective, and the analysis of the relative growth of the spheres was performed using ImageJ 1.5.3. The images were taken at 0 h, 24 h, 48 h, 96 h, and 168 h (1 week) post-treatment.

### 2.10. Statistical Analysis

All data are presented as the means  ±  SEMs from at least three independent experiments. The data groups were compared using two-tailed Student’s *t*-tests or one-way ANOVA followed by Tukey post hoc tests using the GraphPad Prism 6.0 software. Differences between groups were considered statistically significant if *p*  <  0.05. Statistical significance is denoted by asterisks (* *p*  <  0.05; ** *p*  <  0.01; and *** *p*  <  0.001), and non-significant differences are marked as NS.

## 3. Results

### 3.1. O-GlcNAcylation, OGT, and GFAT2 Were Elevated in GB Cells 

**Figure 1:**
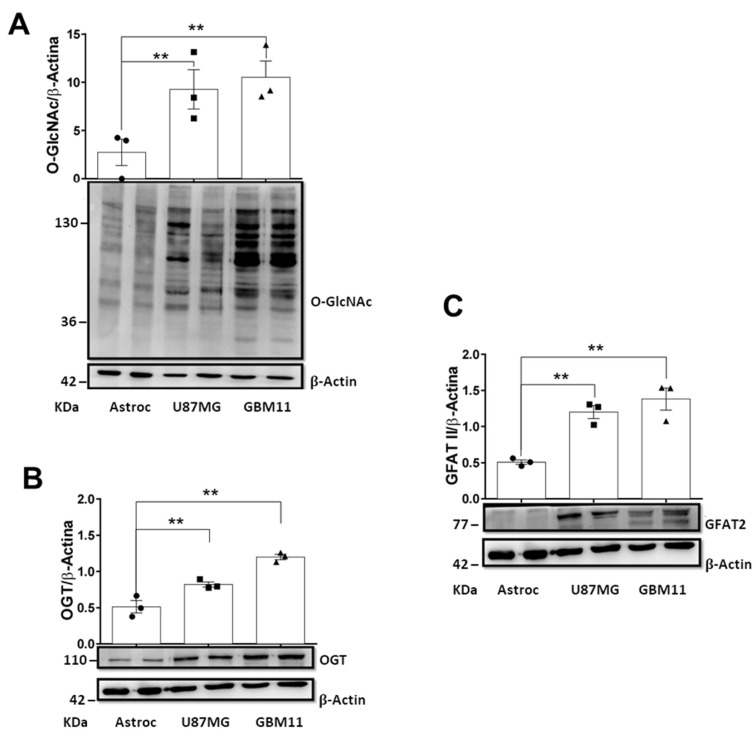
O-GlcNAcylation, OGT, and GFAT2 protein levels were elevated in the glioblastoma cell lines. (**A**) The total O-GlcNAcylation, (**B**) OGT, and (**C**) GFAT2 protein levels were measured by Western blotting in the astrocytes, U87MG, and GBM11 cells. Quantification of the protein levels in each cell line was normalized to β-actin. The data are presented as scatter plot with bar of at least three biological replicates performed. ** *p* < 0.1. Two-Way ANOVA with Tukey’s multiple comparison teste (GraphPad Prism 9). The values shown are expressed as means ± SEMs. The uncropped blots are shown in Supplementary Material.

First, we conducted a comparative analysis of the O-GlcNAcylation levels in two human GB cell lines: U87MG (commercial) and GBM11 (a more malignant cell line isolated from a tumor recurrence). We used human astrocytes as the non-tumoral control. Our results revealed the significant enhancement of O-GlcNAcylation in both GB cell lines with at least a 3.0-fold increase when compared to the astrocytes. Notably, the GBM11 cells exhibited higher O-GlcNAcylation levels than the U87MG cells (Figure 1A). Next, we investigated the relationship between OGT levels and cell malignancy. We observed an increase in OGT expression in the GB cells, and this increase was correlated with cell malignancy, with the GBM11 cells showing higher OGT levels compared to the U87MG cells (Figure 1B). We analyzed the protein levels of GFAT2, the key enzyme involved in the production of UDP-GlcNAc, the donor substrate for OGT. GFAT2 is a major isoform found in the central nervous system. Our analysis revealed significantly higher expression levels of GFAT2 in both GB cell lines compared to the untransformed human astrocytes (Figure 1C). Overall, our findings suggested a potential link between the O-GlcNAcylation machinery and the malignancy of the GB cells.

### 3.2. Elevated O-GlcNAcylation Promoted an Increase in the Number of Viable Cells Not Affecting Cell Cycle Progression in the GB Cells

**Figure 2:**
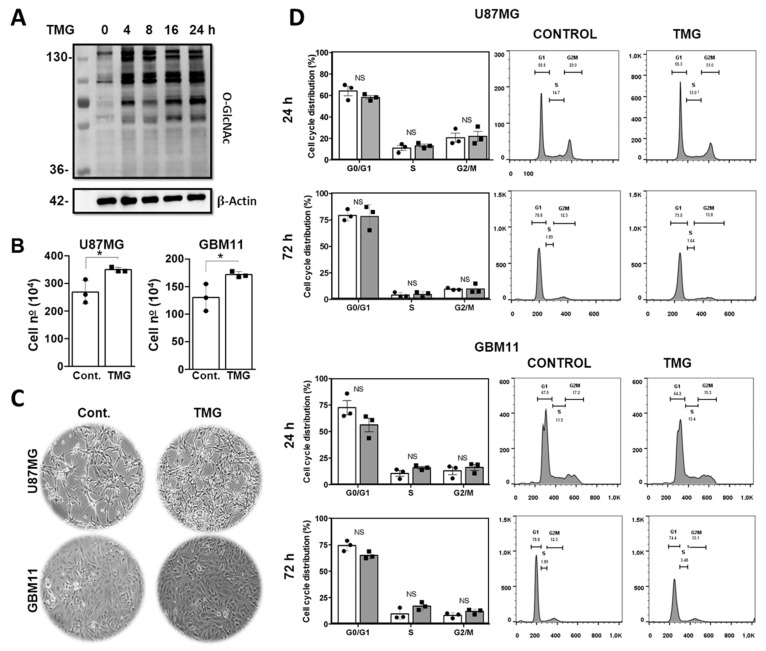
Enhanced O-GlcNAcylation promoted increases in viable glioblastoma cells in the culture without affecting the cell cycle profiles. (**A**) Human glioblastoma cells (U87-MG) were incubated with 1 μM TMG for 4, 8, 16, and 24 h and analyzed for O-GlcNAcylation. β-actin was used as a loading control. Representative image of three independent experiments. (**B**) The U87MG and GBM11 cell lines were incubated for 24 h with 1 μM TMG and submitted to a trypan blue exclusion assay to count the live cells. Graph representing the average of three independent experiments. The values shown are expressed as means ± SEMs. * *p* < 0.05. The statistical test used was the Student’s *t*-test. (**C**) Representative images of the increase in the number of GB cells in the culture after incubation with 1 μM TMG for 24 h compared to the control condition. (**D**) Cell cycle analysis of the U87MG and GBM11 cell lines with or without 1 μM of TMG for 24 and 72 h. A total of 10,000 events were acquired, and there were no cycle changes in either of the GB cell lines. The uncropped blots are shown in Supplementary Material. Representative cell cycle of one experiment and graphs of the percentage of the average of three independent experiments are shown, with the means ± standard errors of the means. The statistical test used was two-way ANOVA. NS, no statistically significant difference. All experiments were performed with at least three biological replicates.

To assess the impact of high levels of O-GlcNAc on the GB cell lines, we used the specific OGA inhibitor TMG [34]. The drug’s efficiency was confirmed by Western blot, showing an increase in O-GlcNAcylation after 4 h of treatment (Figure 2A). Cell viability and proliferation were assessed by the trypan blue exclusion assay. We observed that increased O-GlcNAcylation significantly increased the number of viable cells in the culture in approximately 20% of both the U87MG and GBM11 cell lines (Figure 2B). This effect was further corroborated by phase-contrast microscopy (Figure 2C).The increase in viable cells in the culture induced by TMG motivated us to analyze its impact on cell cycle progression [36]. To investigate this, we synchronized the U87MG and GBM11 cells in the G0 phase through 24 h of serum starvation and subsequently treated them with or without 1 μM TMG. The cells were stained with propidium iodide (PI) and analyzed using flow cytometry. The increase in O-GlcNAcylation did not alter the cell cycle profiles of the GB cells incubated for 24 or 72 h with TMG as compared to the untreated cells (Figure 2D).

### 3.3. O-GlcNAcylation Modulated Autophagy in the U87MG Cells

**Figure 3:**
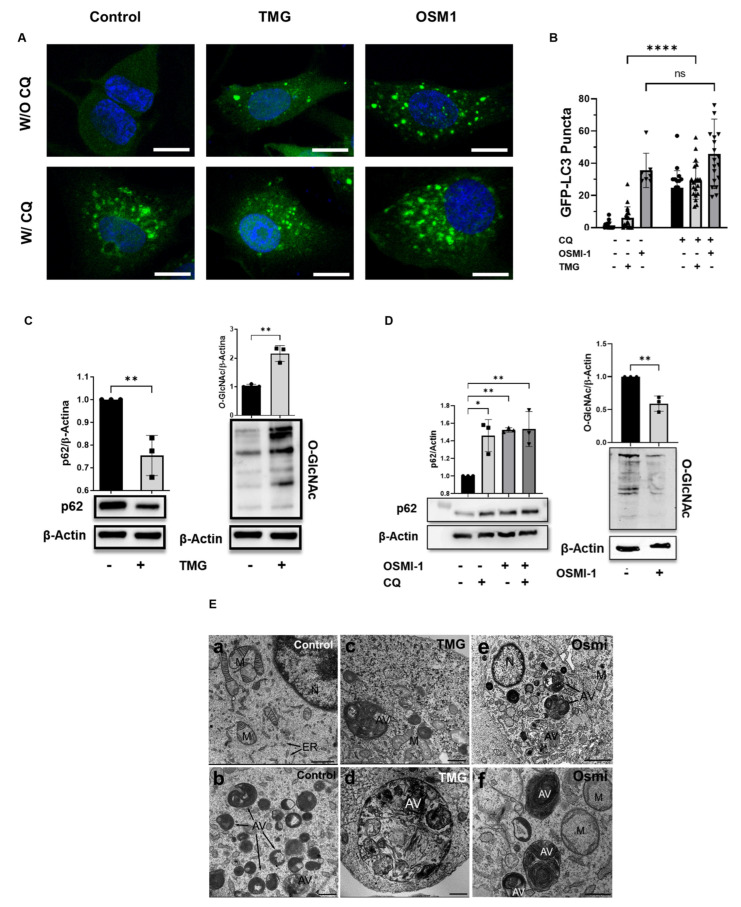
Increased O-GlcNAcylation activated autophagic flux in the GB cell lines while reduced O-GlcNAcylation inhibited autophagy similarly to CQ. (**A**) Representative immunofluorescence images demonstrating the GFP−LC3 puncta in the U87MG cells. Scale bar: 10 μm. (**B**) Quantification of the LC3 puncta average number from the images in (**A**). **** *p* < 0.0001; “ns” indicates non-significant (*p* > 0.05). (**C**) The O-GlcNAcylation levels and expression of the p62 levels in the U87MG cells 24 h after incubation with TMG were determined by an immunoblotting assay. β−actin was used as a loading control. The images are representative of three independent experiments performed. The levels of relative expression (black bars, means ± standard errors of the means, *n* = 3) are shown on the right side of the figure. The statistical test used was the Student’s *t*-test in which ** *p* < 0.01. (**D**) The expression of the total O-GlcNAcylation after incubation of the U87MG cells with Osmi-1 for 24 h and the p62 levels after incubation with CQ, Osmi-1, or cotreatment with Osmi-1 and CQ for 24 h were also determined by an immunoblotting assay. β-actin was used as a loading control. The levels of relative expression (black bars, means ± standard errors of the means, *n* = 3) are shown on the right side of the figure. The statistical test used was the Student’s *t*-test in which * *p*  <  0.05; ** *p*  <  0.01. The uncropped blots are shown in Supplementary Material. (**E**) Qualitative and morphological analysis of the presence of autophagosomes in the U87MG cells incubated in the absence (**a**,**b**) or presence of 1 μM TMG for 24 h (**c**,**d**) or 25 μM Osmi-1 for 24 h (**e**,**f**) using transmission electron microscopy. (**a**) In the control cells, numerous mitochondria were evident without any morphological alterations. The endoplasmic reticulum (ER) profiles and nuclei (N) were also observed. In (**c**–**f**), glioblastoma cells treated with TMG or Osmi-1 showed several autophagic vacuoles (AV). Scale bar: (**a**) 1 µm and (**b**–**f**) 500 nm.

The absence of differences in the cell cycles motivated us to focus on evaluating the autophagy mechanism, which is known to be a crucial cell survival mechanism in glioblastoma cells [37]. To detect autophagy, we employed a well-established technique of counting LC3-II puncta using confocal microscopy [38]. We generated a stable U87MG GFP-LC3-II cell line for counting the LC3 puncta numbers, and then we treated the cells with TMG to increase O-GlcNAcylation or with the OGT inhibitor Osmi-1 [39] to decrease O-GlcNAcylation. Additionally, we used chloroquine to inhibit autophagic flux by impeding the autophagosome-lysosome fusion, thereby increasing the LC3-II puncta [40]. Figure 3A shows that TMG and chloroquine induced increases in the LC3-II points in the cell cytoplasm when compared to the control. This effect was enhanced when TMG and chloroquine were combined (Figure 3B). We observed an increase in the number of LC3-II puncta when the O-GlcNAc was reduced by an Osmi-1 treatment. However, treatment with Osmi-1 combined with CQ displayed no significant increases in the number of LC3 puncta when compared to treatment with Osmi-1 alone, suggesting that both CQ and Osmi-1 were acting in the autophagosome–lysosome fusion (Figure 3B). Another autophagy marker is the p62 protein that accumulates when autophagy is inhibited, and it decreases when autophagy is induced [41]. Thus, cells of the U87MG cell line were incubated with TMG or Osmi-1 for 24 h and subjected to Western blotting assays to evaluate the p62 expression and total O-GlcNAcylation. Figure 3C shows the effect of TMG in increasing the levels of O-GlcNAcylation and in reducing the protein levels of p62. On the other hand, when the cells were incubated with Osmi-1, reductions in the O-GlcNAcylation levels and p62 accumulation were observed (Figure 3D), confirming the inhibitory action of Osmi-1 on autophagy in GB cells. The effect of Osmi-1 on p62 protein levels was similar to the effect of CQ, and the co-treatment continued to promote p62 accumulation as isolated treatments. Furthermore, we performed a morphological analysis of the autophagosomes by transmission electron microscopy (MET). The MET images showed the integrity of the organelles in the cells and the presence of autophagic bodies in the cells treated with TMG and Osmi-1 (Figure 3E). The results supported the notion that O-GlcNAcylation is associated with autophagy regulation, with potential implications for GB cell survival.

### 3.4. Hypo-O-GlcNAcylation Reduced the Viability and Proliferation of the GB Cells but Not the Astrocytes

**Figure 4:**
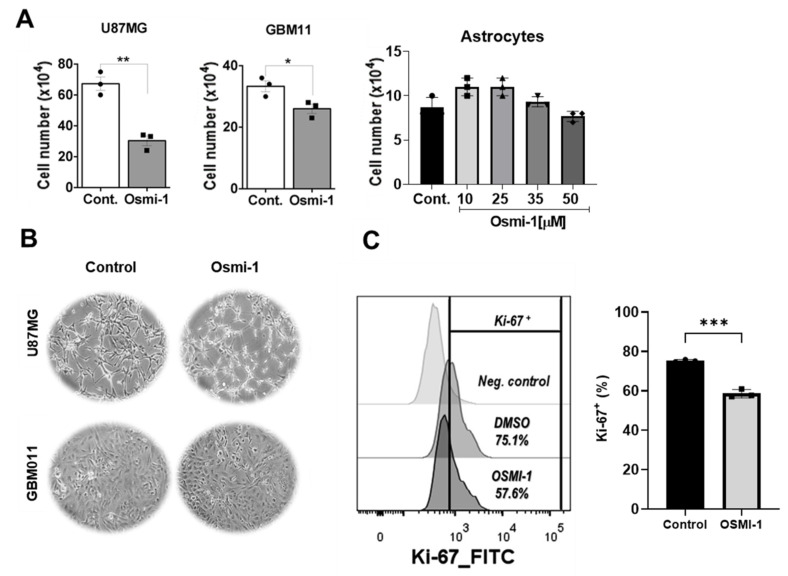
Inhibition of O-GlcNAc inhibited GB cell growth without affecting the astrocytes. (**A**) Trypan blue exclusion assay for the analysis of cell proliferation with cells from the U87MG and GBM11 cell lines incubated with the vehicle solution (DMSO) or 25 µM Osmi-1 for 24 h. The values shown are expressed as means ± SEMs. On the right side of panel, the primary human astrocytes were treated with different concentrations of Osmi-1 (10 μM, 25 μM, 35 μM, and 50 μM) for 24 h and viability was assessed by trypan blue exclusion assays. (**B**) Phase-contrast microscopy with representative images of the U87MG and GBM11 cells treated with DMSO or 25 µM Osmi-1 for 24 h. (**C**) The GBM11 cells were incubated with DMSO or 25 µM Osmi-1 for 24 h and submitted to the Ki-67 flow cytometry staining protocol to analyze cell proliferation. For statistical analysis, two-way ANOVA was used with a Tukey post hoc test in which * *p* < 0.05, ** *p* < 0.01, and *** *p* < 0.001.

Next, we delved into the impact of Osmi-1 treatment on the GB cell lines and the human astrocytes, exploring its effects on cell viability. Treatment with 25 μM Osmi-1 for 24 h reduced the number of living cells in both the GB cell lines, with a higher reduction in the U87MG cells (Figure 4A). Subsequently, we analyzed the impact of Osmi-1 on cell viability in the human astrocytes by subjecting them to increasing concentrations of Osmi-1 (10 μM, 25 μM, 35 μM, and 50 μM) using trypan blue. Notably, even at the highest concentration of 50 μM Osmi-1, there was no reduction in the astrocytes (Figure 4A, right panel). Additionally, we utilized phase-contrast microscopy to assess the cell morphologies of both GB cell lines and the human astrocytes cells treated with the vehicle or 25 µM Osmi-1 (Figure 4B). These findings collectively highlighted the differential impacts of Osmi-1 on the GB cells and the human astrocytes, suggesting its potential as a selective therapeutic agent for treating glioblastoma. Further, by employing a cytometry assay with Ki-67 labeling, we evaluated the effect of Osmi-1 on the cell proliferation of the GB cells (Figure 4C). The data showed that hypo-O-GlcNAcylation decreased the percentage of cycling cells in the population of GB cells. 

### 3.5. O-GlcNAcylation Inhibition Enhanced GB Sensitivity to TMZ Chemotherapy 

**Figure 5:**
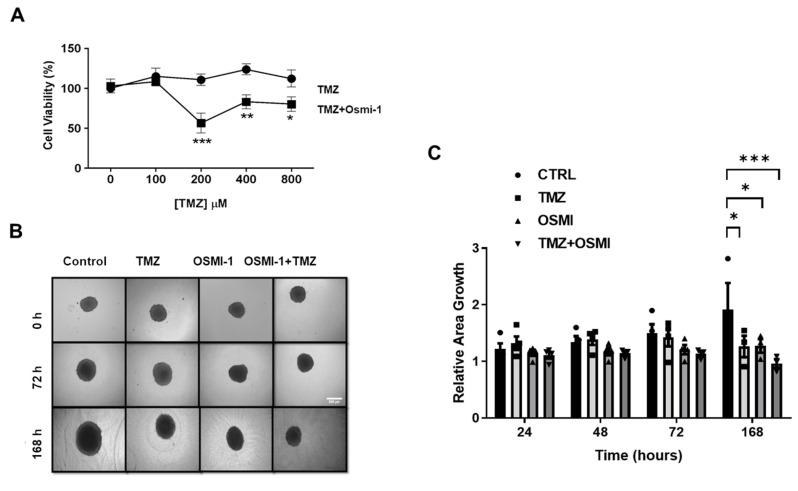
Inhibition of O-GlcNAc inhibited GB cell growth and sensitized them to chemotherapy. (**A**) Through an MTT assay, the percentage of viable cells (*y*-axis) in the U87MG lineage was analyzed within 24 h under treatment with different concentrations of TMZ associated (or not) with 25 μM Osmi-1 (*x*-axis). Graph representing the means and standard errors of three independent experiments. Each concentration was evaluated in five replicates. For the statistical analysis, two-way ANOVA was used with a Tukey post hoc test in which * *p* < 0.05, ** *p* < 0.01, and *** *p* < 0.001. (**B**) The U87MG spheroids were incubated (or not) (DMSO) with Osmi-1, 200 µM TMZ, or a combination of Osmi-1 and TMZ for 0, 24, 48, 72, and 168 h, with analyses at every 24 h of treatment. The experiments were carried out as at least three times with three replicates each. Representative images of the spheroids treated (or not) at 0, 72, and 168 h. Scale bar: 300 µm. (**C**) Graph representing the means and standard errors of B. For the statistical analysis, two-way ANOVA was used with a Tukey post hoc test in which * *p* < 0.05, ** *p* < 0.01, and *** *p* < 0.001.

The impact of hypo-O-GlcNAcylation on GB cells with no effects on the human astrocytes prompted us to assess the effect of Osmi-1 in combination with TMZ, the most-used chemotherapeutic for treating GB. Firstly, the cells were treated with increasing concentrations of TMZ (0, 100, 200, 400, and 800 μM), associated (or not) with 25 μM of Osmi-1 for 24 h. Although no effect was observed upon TMZ incubation, co-treatment with Osmi-1 and TMZ significantly reduced the cell viability at 200 μM (Figure 5A). Three-dimensional spheroids have emerged as an appropriate system for drug screening [42]. Given that GBM11 does not form spheroids, the impact of the combined treatment of 25 µM Osmi-1 and 200 µM TMZ was tested on the U87MG cells. The cells were cultured for 4 days to form spheroids in agarose-coated plates and further treated (or not) with Osmi-1, TMZ, or both drugs. Then, the spheroid area was analyzed at time intervals of 0, 24, 48, 72, and 168 h (1 week). Figure 5B shows that the Osmi-1 treatment tended to stagnate spheroid growth after 24 h of incubation, while treatment with TMZ was only able to reduce the size of the spheroids after 1 week of treatment. The combined Osmi-1 and TMZ treatment resulted in stagnated spheroid growth after 24 h, with a greater reduction observed after 1 week (Figure 5C). Collectively, these results indicated that reducing O-GlcNAcylation hampered the growth of the GB spheroids and sensitized these cells to TMZ chemotherapy, suggesting the potential of this combination as a promising therapeutic approach in GB treatment. 

### 3.6. Synergistic Effects of Osmi-1 and TMZ in GB Apoptosis

**Figure 6:**
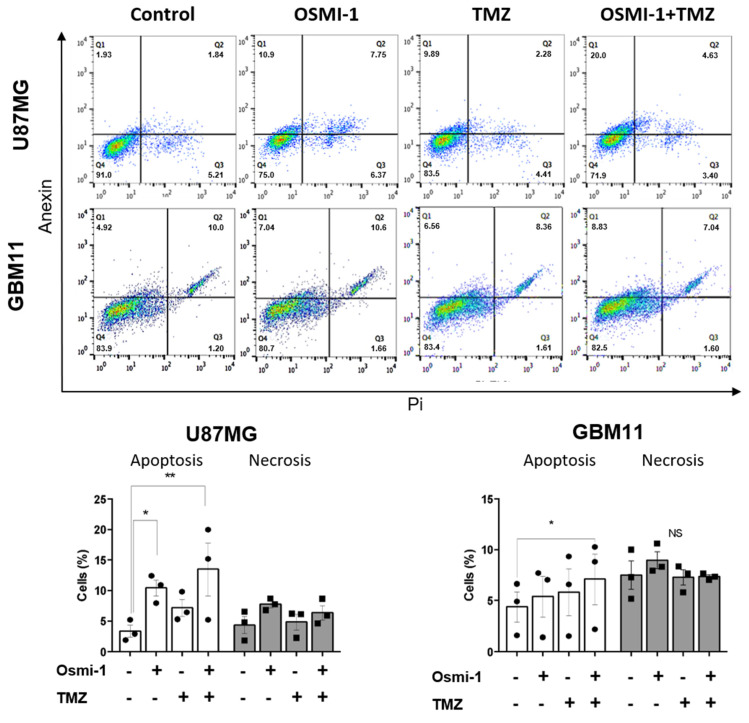
Pharmacological inhibition of OGT in combination with chemotherapy induced GB cell death by apoptosis. Representative plot of the flow cytometric analysis data and graphic representation of three independent experiments. Apoptosis was analyzed by flow cytometry. For the statistical analysis, Two−Way ANOVA was used with a Tukey post hoc test in which * *p* < 0.05 and ** *p* < 0.01. NS, no statistically significant difference.

The effects of Osmi-1, TMZ, and their combined treatment on apoptosis and necrosis were also analyzed. We assessed cell death by apoptosis or necrosis by using PI and Annexin V labeling. Annexin V labeling occurs when a cell exposes phosphatidylserine on its surface, indicating cell death by apoptosis. Approximately 2–3% of the U87MG control cells and approximately 9% of the cells incubated with 200 μM TMZ were positive for Annexin labeling. The combination of Osmi-1 and TMZ further heightened the apoptotic response, with approximately 15% of the cells showing cell death through apoptosis (Figure 6). Regarding the GBM11 cell line, a significant increase in cell death was observed only when Osmi-1 and TMZ were co-administered, endorsing the synergistic effect of the treatments (Figure 6). These results emphasized the potential of combining Osmi-1 with TMZ as a strategy to enhance apoptotic responses in GB cells, suggesting a promising avenue for novel therapeutic interventions.

## 4. Discussion

**Figure 7:**
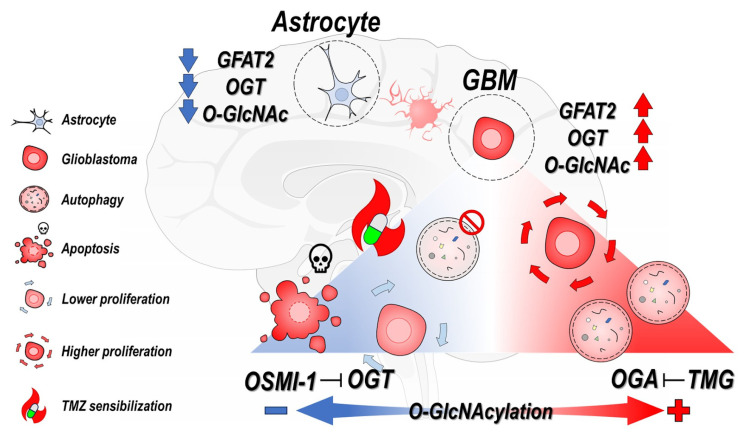
Effects of O-GlcNAcylation modulation in human glioblastoma cells. Glioblastoma cells have higher levels of total O-GlcNAcylation and higher levels of protein expression of OGT and GFAT 2 compared to human astrocyte cells. In the image is also demonstrated the opposite effects of pharmacological inhibitors of the enzymes OGT (Osmi-1) and OGA (TMG) on glioblastoma cells. The effects of hypo O-GlcNAcylation (Blue) and hyper O-GlcNAcylation (Red) were also illustrated according to the image caption on the left side of the figure.

GB, also referred to as a grade IV astrocytoma, is one of the most aggressive and resistant-to-treatment tumors. Patients affected by this pathology have an average life expectancy of just 15 months from diagnosis of the disease to death, indicating the urgency for the development of new treatments [3,11]. Growing evidence has demonstrated that altered metabolism is a promising target for developing therapies against GB [11,12,43]. Many recent efforts have aimed to understand the molecular mechanism involved in the metabolic reprogramming observed in cancer cells. Emerging data have shown that O-GlcNAcylation acts in tumor progression and metabolism control [44,45,46,47]. The brain presents with high O-GlcNAcylation levels, which suggests its importance in the central nervous system [48,49]. In this work, we showed that two human GB cell lines (U87MG and GBM11) had higher O-GlcNAcylation levels than non-tumoral human astrocytes (Figure 7). Increased O-GlcNAcylation levels were accompanied by higher levels of OGT, where the GBM11 cells showed greater expression levels when compared with the U87MG cells. The higher O-GlcNAcylation and OGT levels observed in GBM11, cells originated from a tumor recurrence process, might indicate their roles in tumorigenesis. In fact, increases in O-GlcNAcylation levels have been observed in most of the tumors studied [50,51,52,53], and they are associated with the tumorigenesis and proliferation of cancer cells [46,54,55]. Corroborating our results, a recent work demonstrated that the U87MG and T98G human glioblastoma cell lines and tissue samples from patients with GB grade IV had higher protein levels of O-GlcNAcylation and OGT compared to healthy astrocyte cells [53]. In addition, they showed the importance of OGT for GB cell growth in vitro and in vivo by regulating acetate metabolism [53]. Here, we also analyzed the expression levels of GFAT, the rate-limiting enzyme of HBP. There are two GFAT isoforms in mammals, GFAT1 and GFAT2, which are expressed in different tissues [56]. GFAT1 is described as widely expressed while GFAT2 is significantly expressed in the nervous system and some tumor types [57,58,59,60]. Here, we showed a higher expression of GFAT2 in the GB cell lines (U87MG and GBM11) than astrocyte cells, suggesting activation of HBP and supporting the increase of O-GlcNAcylation levels observed in GB cells (Figure 7). 

In addition, we showed that the GB cell lines incubated with the pharmacological OGA inhibitor TMG had increased cellular proliferation (Figure 7). In accordance with our data, the elevation of O-GlcNAcylation in gastric cancer cell lines promoted an increase in cell proliferation [61]. Similarly, the incubation of anaplastic thyroid carcinoma cells with TMG for 48 h caused increases in cell proliferation and viability [62]. Furthermore, we showed that the inhibition of OGA increased proliferation and altered the composition of the GB secretome [63]. In fact, O-GlcNAcylation contributes to the regulation of DNA replication, mitosis, and cytokinesis [36]. Thus, in this work, we evaluated the effect of TMG on the cell cycle of GB cells. Surprisingly, no changes in the cell cycle were detected in the GB cells incubated with TMG. On the other hand, increase of O-GlcNAcylation by TMG treatment led to increases in the S and G2/M phases in gastric cancer cells [61]. 

Studies using different models have shown that O-GlcNAcylation interferes with autophagy mechanisms [20,22,29,64,65]. In fact, both autophagy and O-GlcNAcylation play pivotal roles in regulating cellular fate, responding to sensory and nutritional cues. In our experiments, TMG treatment induced autophagy, especially when combined with chloroquine, which corroborated previous findings in neuroblastoma cells, rat primary neurons, and astrocytes [66]. Understanding the molecular mechanisms underlying these processes holds promise for developing therapeutic strategies that target autophagy, which is crucial for the metabolism of nervous system cells and for glioblastoma cell resistance to TMZ chemotherapy and radiotherapy [66,67]. Our results suggested that autophagy may be the mechanism responsible for the increased proliferation observed when the GB cells were treated with TMG. On the other hand, the reduction in O-GlcNAcylation levels by Osmi-1 treatment generated a high level of GFP-LC3 puncta, with no significant difference observed for co-treatment with chloroquine. This suggested that both drugs act by inhibiting autophagosome–lysosome fusion. Additionally, the results involving the p62 levels supported the hypothesis that TMG induces autophagy by decreasing p62 levels, whereas Osmi-1 inhibited autophagosome–lysosome fusion, leading to increased p62 levels. Previous works have demonstrated that both increases [66] and reductions in O-GlcNAcylation levels can contribute to autophagy [29,68]. Indeed, different works have already demonstrated that autophagy favors the survival and therapeutic resistance of GB [28,69,70,71]. The inhibition of autophagy by Osmi-1 treatment has motivated us to further analyze its effect on apoptosis and necrosis, which could provide crucial insights into potential therapeutic approaches targeting GB cell survival pathways.

We demonstrated that both GB of the cell lines (U87MG and GBM11) treated with 25 μM of Osmi-1 for 24 h presented with reduced numbers of viable cells. In contrast, no significant changes in astrocyte viability were detected even when the cells were incubated with 50 μM of Osmi-1. These results indicated the potential therapeutic of OGT inhibition. A recent study showed that OGT deficiencies induced the activation of astrocytes in vivo and in vitro [72]. In our work, GB cells incubated with Osmi-1 and TMZ suffered reduced proliferation and viability when compared with cells treated only with TMZ, indicating that reductions in O-GlcNAc increase sensitivity to chemotherapeutics. In addition, we cultivated U87MG in a 3D culture system. This type of culture simulates tumor characteristics, such as senescence, hypoxia, and anti-apoptotic behavior [73,74,75]. In this work, the treatment of spheroids with TMZ only reduced the sizes of the spheroids after one week of treatment. In contrast, the treatment with Osmi-1 promoted the stagnation of spheroid growth after 24 h of incubation, indicating a fast effect. The combined treatment of Osmi-1 and TMZ was more effective than the isolated treatments since we were able to significantly reduce the sizes of the spheroids after a 1-week period of incubation. Treatment with Osmi-1 suppressed the number of spheres in the HCT116 and HCC827 cells [76]. Liu and colleagues demonstrated that hepatocarcinoma and breast cell lines became more sensitive to the chemotherapy drug Doxorubicin (Dox) increasing apoptosis after treatment with Osmi-1 [77]. Numerous studies have shown that the reduction in O-GlcNAcylation levels by treatment with Osmi-1 promoted decreased cell proliferation in vitro (or reduced growth of the tumor mass) [76,78,79].

## 5. Conclusions

Our study demonstrated that GB cell lines exhibit significantly higher levels of O-GlcNAcylation, OGT, and GFAT compared to non-tumoral human astrocytes. Hyper-O-GlcNAcylation promoted increased cell proliferation and autophagy in GB cells. In contrast, hypo-O-GlcNAcylation led to autophagy inhibition and reduced cell proliferation in both the 2D and 3D models, and it induced cell death by apoptosis while not affecting the viability and proliferation of the control astrocytes. Collectively, our findings suggested that hyper-O-GlcNAcylation may contribute to GB’s aggressiveness and chemotherapy resistance. Conversely, the reduction in O-GlcNAcylation levels indicated that targeting OGT could be a promising therapeutic approach, or it could be used in combination with the chemotherapeutic agent TMZ to improve the treatment of GB. These insights provide a foundation for further exploring O-GlcNAcylation as a potential therapeutic target in GB treatment, with the ultimate goal of developing more effective treatment strategies for this aggressive brain tumor.

## Data Availability

The data presented in this study are available in this article.

## Supplementary Materials

The following supporting information can be downloaded at: https://www.mdpi.com/article/10.3390/cancers15194740/s1.
