# Supplementary material for: Inhibition of O-GlcNAcylation Reduces Cell Viability and Autophagy and Increases Sensitivity to Chemotherapeutic Temozolomide in Glioblastoma
Source: Cancers (Basel). 2023 Sep 27;15(19):4740. doi: 10.3390/cancers15194740 (PMC10571858; doi:10.3390/cancers15194740)
Supplement: Supplementary file 1 [file cancers-15-04740-s001.zip › cancers-2592550-supplementary.pptx]

## Slide 1
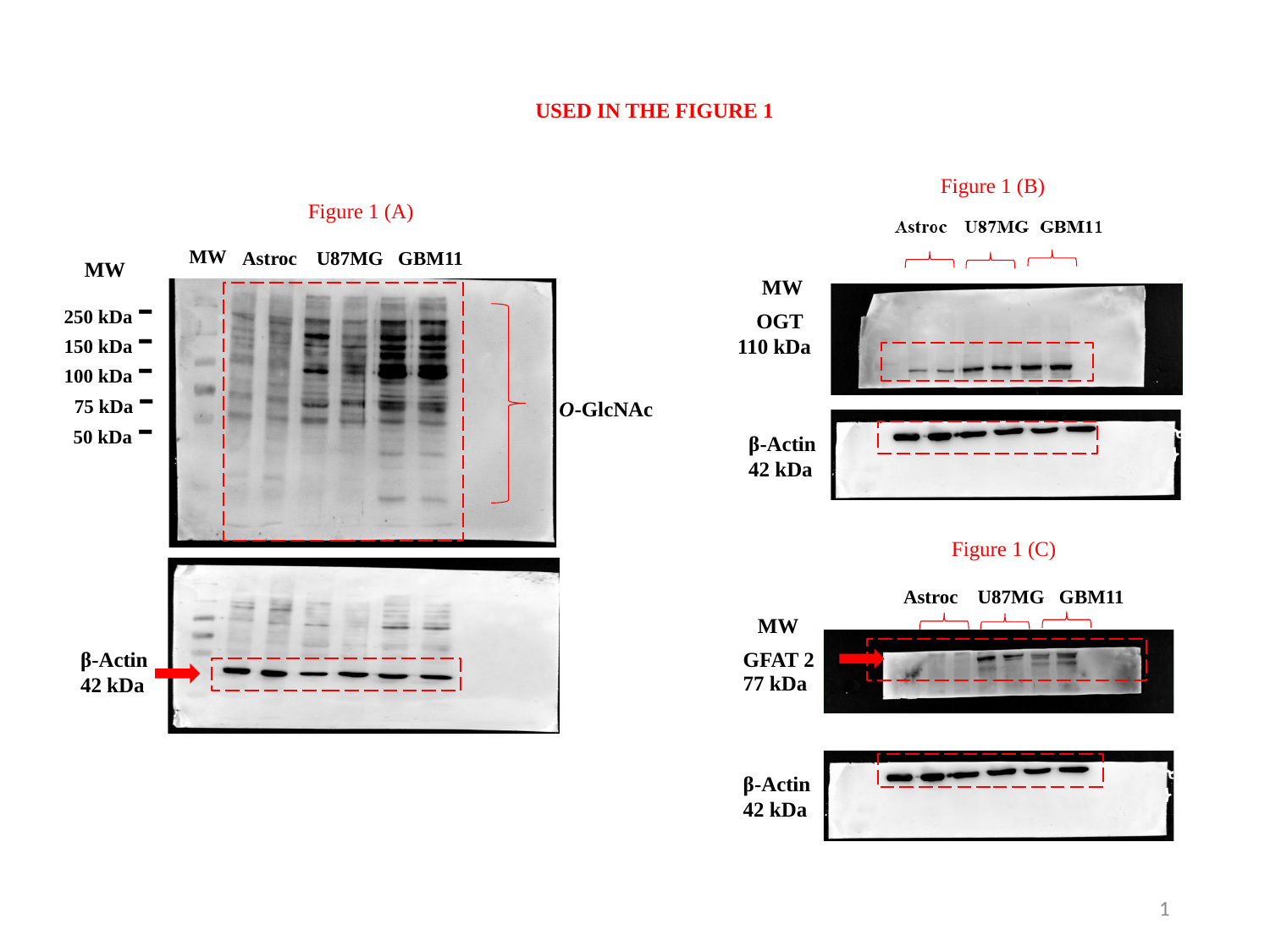

USED IN THE FIGURE 1
Figure 1 (B)
Figure 1 (A)
Astroc U87MG GBM11
250 kDa -
MW
MW
150 kDa -
MW
100 kDa -
OGT
75 kDa -
110 kDa
50 kDa -
O-GlcNAc
β-Actin
42 kDa
Figure 1 (C)
Astroc U87MG GBM11
MW
β-Actin
42 kDa
GFAT 2
77 kDa
β-Actin
42 kDa
1

## Slide 2
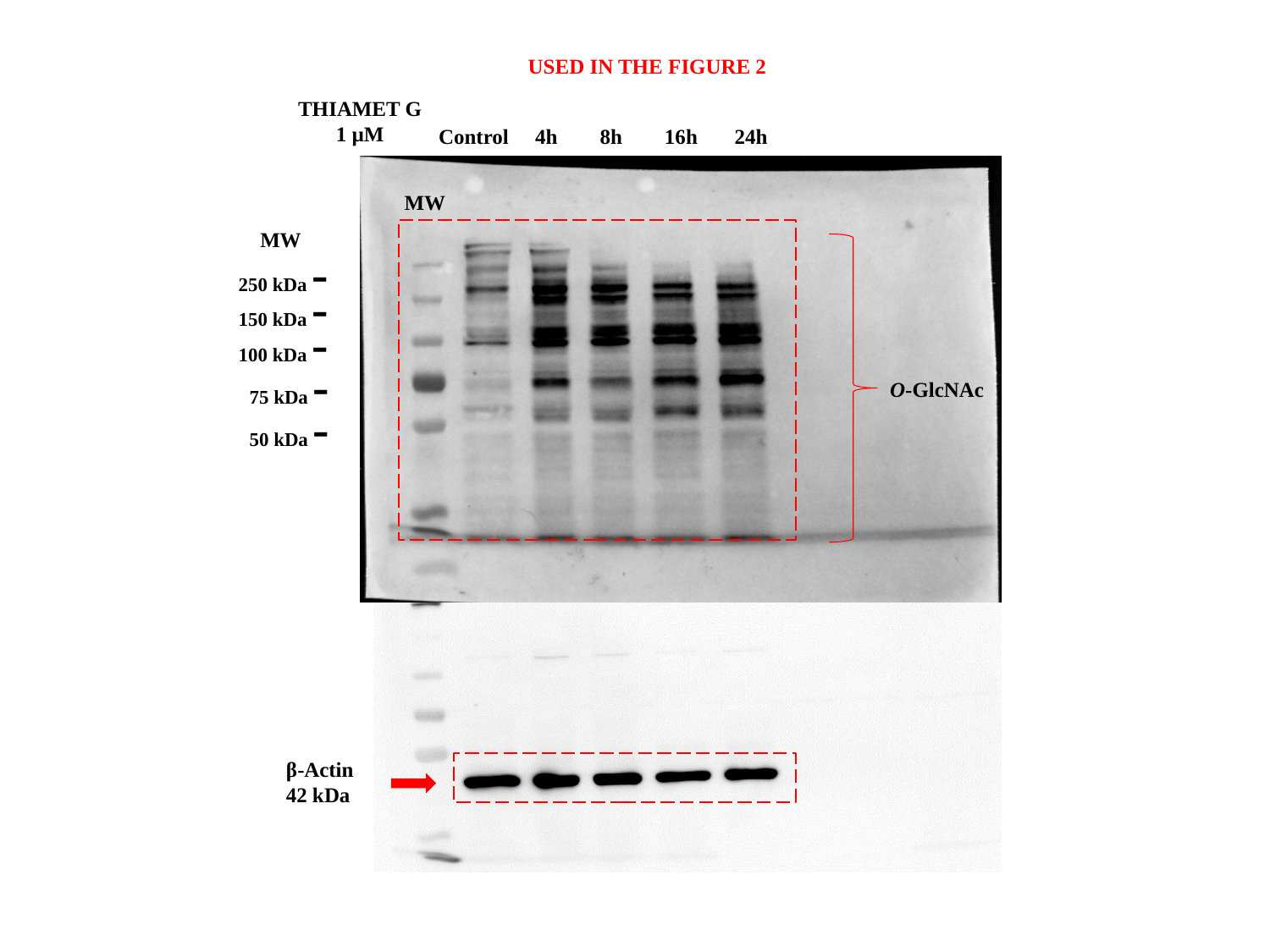

USED IN THE FIGURE 2
THIAMET G
1 µM
 Control 4h 8h 16h 24h
MW
250 kDa -
MW
150 kDa -
100 kDa -
75 kDa -
50 kDa -
O-GlcNAc
β-Actin
42 kDa

## Slide 3
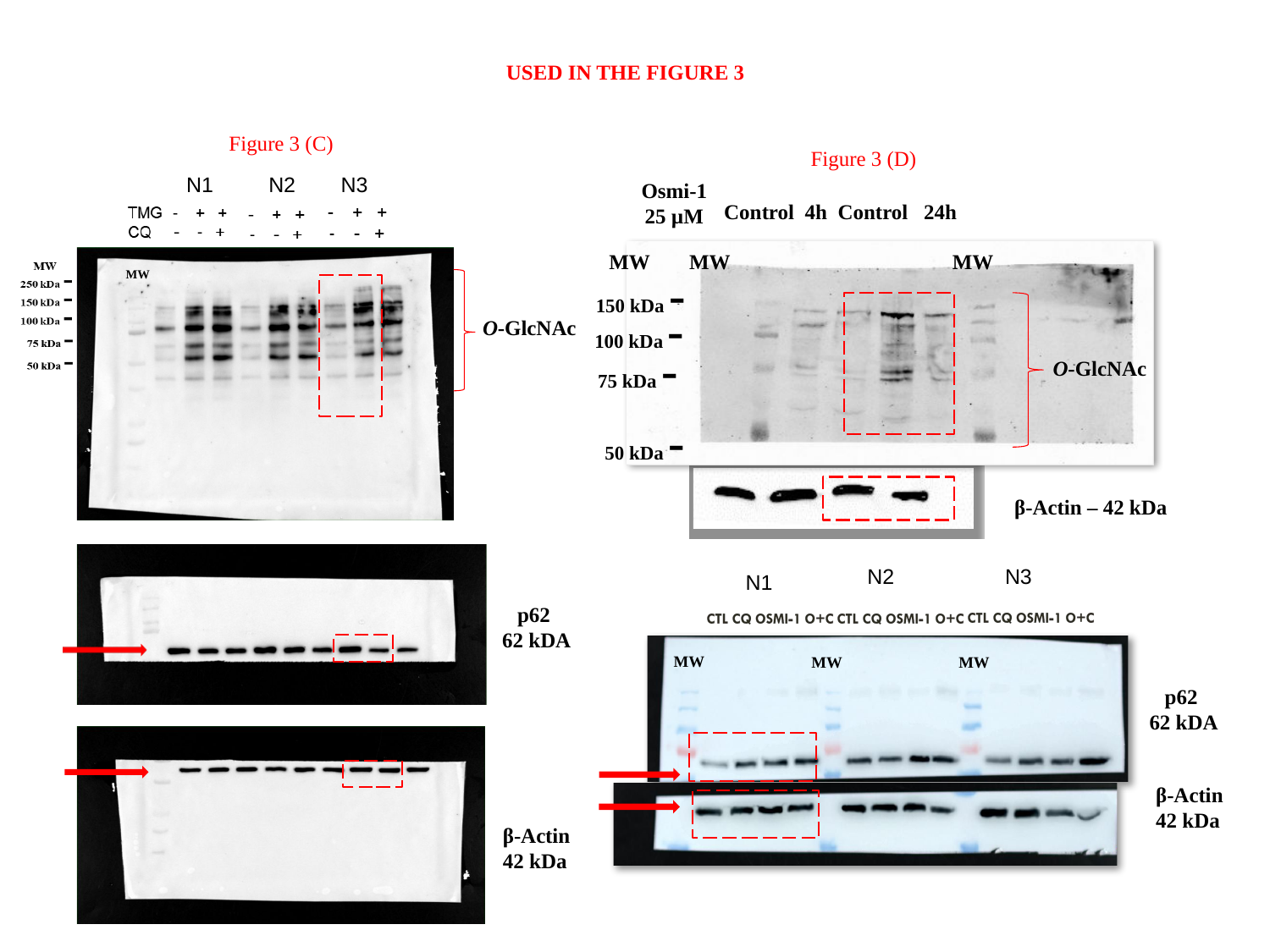

USED IN THE FIGURE 3
Figure 3 (C)
Figure 3 (D)
N2
N1
N3
Osmi-1
25 µM
 Control 4h Control 24h
150 kDa -
MW
100 kDa -
75 kDa -
50 kDa -
MW
MW
MW
O-GlcNAc
O-GlcNAc
β-Actin – 42 kDa
N2
N3
N1
p62
62 kDA
MW
MW
MW
p62
62 kDA
β-Actin
42 kDa
β-Actin
42 kDa
